# How the study of networks informs knowledge translation and implementation: a scoping review

**DOI:** 10.1186/s13012-019-0879-1

**Published:** 2019-03-27

**Authors:** Stephanie M. N. Glegg, Emily Jenkins, Anita Kothari

**Affiliations:** 1 Rehabilitation Sciences, The University of British Columbia, 212 - 2177 Wesbrook Mall, Vancouver, BC V6T 1Z3 Canada; 2 Therapy Department, Sunny Hill Health Centre for Children, 3644 Slocan Street, Vancouver, BC V5M 3E8 Canada; 3 BC Children’s Hospital Research Institute, 938 West 28th Ave, Vancouver, BC V5Z 4H4 Canada; 4 School of Nursing, The University of British Columbia, T201-2211 Wesbrook Mall, Vancouver, BC V6T 2B5 Canada; 5 School of Health Studies, Western University, Arthur and Sonia Labatt Health Sciences Building, Room 222, London, ON V6A 5B9 Canada

**Keywords:** Knowledge translation, Evidence-based practice, Social network analysis, Information flow, Scoping review, Network, Implementation

## Abstract

**Background:**

To date, implementation science has focused largely on identifying the individual and organizational barriers, processes, and outcomes of knowledge translation (KT) (including implementation efforts). Social network analysis (SNA) has the potential to augment our understanding of KT success by applying a network lens that examines the influence of relationships and social structures on research use and intervention acceptability by health professionals. The purpose of this review was to comprehensively map the ways in which SNA methodologies have been applied to the study of KT with respect to health professional networks.

**Methods:**

Systematic scoping review methodology involved searching five academic databases for primary research on KT that employed quantitative SNA methods, and inclusion screening using predetermined criteria. Data extraction included information on study aim, population, variables, network properties, theory use, and data collection methods. Descriptive statistics and chronology charting preceded theoretical analysis of findings.

**Results:**

Twenty-seven retained articles describing 19 cross-sectional and 2 longitudinal studies reported on 28 structural properties, with degree centrality, tie characteristics (e.g., homophily, reciprocity), and whole network density being most frequent. Eleven studies examined physician-only networks, 9 focused on interprofessional networks, and 1 reported on a nurse practitioner network. Diffusion of innovation, social contagion, and social influence theories were most commonly applied.

**Conclusions:**

Emerging interest in SNA for KT- and implementation-related research is evident. The included articles focused on individual level evidence-based decision-making: we recommend also applying SNA to meso- or macro-level KT activities. SNA research that expands the range of professions under study, examines network dynamics over time, extends the depth of analysis of the role of network structure on KT processes and outcomes, and employs mixed methods to triangulate findings, is needed to advance the field. SNA is a valuable approach for evaluating key network characteristics, structures and positions of relevance to KT, implementation, and evidence informed practice. Examining how network structure influences connections and the implications of those holding prominent network positions can provide insights to improve network-based KT processes.

Contributions to the literature

- This review synthesizes the KT literature employing a social network analysis (SNA) approach to the study health professionals, to demonstrate the utility of SNA for advancing KT science
- Also summarized is the use of theory in this SNA research to demonstrate the fit of SNA with theoretical approaches used in KT research, including diffusion of innovation and complexity theory
- This article acts as a reference tool for those considering applying a SNA lens to their KT research, to support the design of SNA-specific research questions, methodological approaches, and measures

## Background

The “science” of knowledge translation (KT), namely the study of the processes, determinants, and outcomes of KT and evidence informed practice (EIP) efforts [1], is of high interest to researchers because of the significant challenges that exist in getting research into practice. To date, KT scientists have targeted their efforts primarily at identifying the key processes of KT, understanding the influences on these processes, and evaluating the effectiveness of various strategies to support them [2–5]. However, identifying the specific mechanisms by which KT strategies, particularly complex multi-faceted ones, have been effective requires additional research. Furthermore, limited information is available to clinicians and researchers about the role of social relationships and network connections in facilitating KT [6].

Social network analysis (SNA) is a research paradigm concerned with the patterns of connections (i.e., ties) between actors (i.e., people or entities) within an interconnected group or network, and how this “social structure” impacts outcomes of interest [7]. Key SNA terms that provide the context for this review are defined in Table 1, along with their application to the study of KT. Actors may be individuals, organizations, countries, or other entities; ties reflect the connections or linkages between them [7]. The structural characteristics of both whole and individuals’ networks can be studied. Included with the paradigm are theories, such as network, graph, diffusion, and social influence theories, and a set of methodologies that can be applied across a range of substantive problems. Visualization, or mapping, and the use of network descriptive statistics can provide a visual and empirical basis for comparison across networks, including identifying important strengths, gaps, or differences between networks that merit further exploration through qualitative means. Statistical and computational modeling can also be used to explain and to predict network-related phenomena, and to simulate the complexities inherent in network dynamics.

**Table 1:** Social network analysis (SNA) terms and their implications for knowledge translation

SNA term (frequency count)	Definition	Implication for KT
Network	An interconnected group of actors (e.g., people, organizations) [7]	Provides the social context within which KT occurs
Actor	A point (node) in a network that represents an individual, organization or entity connected to other actors (through ties) [7]	Represents the people, teams, or organizations involved in KT processes
Tie (2)	The relations or connections among actors in the network [79]	Represents the interactions, collaborations, or relationships involved in KT Measures one- versus two-way communication, advice seeking, collaboration, etc. [7]
Dyad	Pairwise relations between actors [7]	Represents one of three levels of analysis for social network data (the others being individual node-level and whole network level) [7]
Centralization		
Whole network centralization (3)	Extent to which interconnections are unequal across the network [21] (i.e., concentrated around one or more central individuals) [7]	Thought to enhance ease of knowledge sharing and to promote standard practices of existing protocols [80]. Decentralization may support new innovations, but lead to mixed messaging and decreased clarity because of multiple information sources [72]
Centrality		
Degree centrality (3)	# of direct ties (connections) of an actor	Seen as an indicator of visibility [81], prestige [39] or power [79] resulting from lots of direct contact to many others
Indegree centrality (10)	# of individuals who send (identify) ties to an actor	Considered an index of importance [28] power or influence [40]
Outdegree centrality (5)	# of direct ties an actor sends (identifies) to others [33]	Used to quantify access to resources through colleagues, exposure to evidence and others’ practices; positively associated with EIP use [33]
Betweenness centrality (4)	Extent to which an individual is tied/connected to others who are not connected themselves [40]	Used as a proxy for control of KT processes [39]; high values reflect a favorable position (e.g. brokering potential) [40] for information flow or power [79]
Flow betweenness centrality (3)	How involved an actor is in all of the paths or routes between all other actors (not just those representing the shortest paths) [79]	Used to determine contributions of individuals toward team decision-making; provides insights into structural hierarchy [33] Used as a proxy for ease of bypassing the core individuals in the network [39, 79]
Closeness centrality (2)	Proportion of actors that can be reached in one or more steps [79]	Proxy for degree of access to information [39] or efficiency in communicating with the network (relative reach) [7]
Bonacich centrality (1)	Extent to which an actor is tied to others, weighted according to the centrality (e.g., popularity, importance) of those to whom the actor is tied/connected [79]	Proxy for power or hierarchy within a network; may help to identify network fragmentation/brokering opportunities [14]
Hubs and authorities centrality (1)	The structural prominence of individuals within a core-periphery structured network [32]	Proxy for importance [32]
Tie characteristics		
Tie strength (7)	Value associated with a tie/connection, e.g., frequency of contact, emotional intensity, duration of connection, etc. [7]	Weak ties thought to increase access to new information/opportunities; strong ties seen as required for innovation implementation [82]
Tie homophily (includes external-internal or EI index) (13)	Similarity of connected actors/nodes on a given attribute [7]	Similarities among people create conditions for social contagion (individuals may be more likely to modify their behaviors/attitudes to match those around them) [67, 83]
Tie hierarchy (1)	Connections between actors dissimilar in their status (e.g., according to profession, leadership or power position) [7]	Hierarchy may be a barrier to innovation adoption (e.g., lack of interest from above/resistance from below [29]
Tie reciprocity (8)	The extent to which directional ties to actors are reciprocated (i.e., are bi-directional) [79]	Reciprocity may reflect greater stability or equality (versus hierarchy) [79]
Euclidian distance (1)	A measure of the dissimilarity between the tie patterns of each pair of actors in the network [79]	Can be used to identify key people by their dissimilarity to others (e.g., who has the most research productivity relative to their connected peers) [28] (as a proxy of influence)
Density		
Whole network density (8)	An index of the proportion of existing ties relative to all possible ties in a network [79]	Proxy for efficiency of information flow [79], solidarity [84], or cohesiveness within a network [21]
Ego network density (2)		
Subgroups		
Components/isolates (3)	Portions of the network that contain actors connected to one another, but disconnected from actors of other subgroups [79]	Subgroups and isolates can be targeted to increase connectedness, share information, or mobilize action
Cliques (1)	Maximum # of actors who share all possible connections among themselves [79]	Can describe paths for fostering awareness and adoption of interventions [23]
Clusters (4)	Dense sets of connections in a network [79]	Identifying attributes that influence clustering helps understand KT-related behaviors, such as information seeking (e.g., experts; same department) [36, 41]
Network roles and positions		
Brokers (1)	Actors holding bridging positions in a network (i.e., play a role in connecting subgroups) [79]	Can leverage brokers’ positions for efficient KT by leveraging their tie paths/connectedness [36, 37, 79]
Coreness/Core-periphery index (2)	The core of a network represents the maximally dense area of connections, whereas the periphery represents (to the maximum extent possible), the set of nodes without connections within their group [79]	Power/influence at the core [39]. The most active EIP practitioners may be found at periphery [32]
Structural equivalence (2)	When two actors/nodes have the same relationships to all other nodes in the network—they can be substituted without altering the network [79]	These positions may generate social pressure within a network [24, 25]
Structural holes/constraint (ego network) (2)	Structural holes: absent ties in a network that limit exchange between actors; constraint: degree to which an actor is tied to others who are themselves connected [79]	Inequality among actors can be identified and targeted through KT interventions; may have implications for EIP adoption [31] (e.g., many ties may restrict one’s actions/capacity) [79]
Transitivity/network closure (i.e., network structure related to triads)		
Alternating k-stars (4)	The tendency of actors to create ties [29]	Used as an indicator of hubs within a network [37] or the tendency to share/exchange knowledge [29]
Alternating k-triangles/transitive triads and/or non-closure structures (5)	The extent to which sets of 3 actors form patterns of connections that create larger “clumps” within the network [29, 79]	Assesses tendency to build relationships outside of one’s local group—access to new knowledge [29]
Cyclic closure (1)	The tendency for transitive triads (sets of three actors in which two ties exist) to lead to reciprocal ties within that triad [27]	Cyclic closure thought to reflect non- hierarchical knowledge exchange, which is more effortful to maintain and therefore less likely to be seen in knowledge sharing networks [27]
Alternating independent two-paths (2)	Assesses the conditions required for transitivity (i.e., ties that form between each pair of actors in a set of three actors) [29]	Can determine the extent to which actors tend to build small, closed, non-hierarchical connections that limit broader access to new information [29]

*SNA* social network analysis, *KT* knowledge translation

SNA offers an alternate perspective to behavior change theory-based approaches prevalent in KT science [5]. These latter approaches focus on individual-level factors influencing behavior change, often from a social cognition perspective [5]. Conversely, SNA proposes a network-level perspective that examines how connections among individuals or entities, and the nature of the associated interactions, influence an outcome (e.g., accessing or sharing evidence, changing practice behaviors based on evidence). The paradigm respects the socially driven nature of innovation uptake, and the value inherent in examining not only the processes involved in KT but also the social structures and characteristics of the networks of relationships within which KT occurs. Examples of network-related KT processes that can be examined from a SNA lens include one-way versus two-way exchange of information, the timing and prediction of evidence uptake by different types of individuals, the influence of specific types of people on behavior change, individuals’ capacity for change based on their positions in the network, gaps in the flow of or access to information or resources required for evidence use, and testing the effectiveness of strategies to address gaps or inefficiencies identified in the network.

Recent systematic reviews on SNA in health care have focused on quality and patient safety initiatives [8, 9], on a single profession (i.e., nursing) [10, 11], or on only select network properties (e.g., the study of brokers) [12]. Some reviews focus on conditions (e.g., obesity networks) [12] to explore possible network interactions for potential treatments. Given the complex and interprofessional nature of health care practice, a study of the full breadth of health professions and network properties is required. Furthermore, some of these reviews included non-health care literature (e.g., from television production and corporate business contexts) [13], or neglected to include social sciences databases in which most SNA journals are indexed. The existing broad reviews of health professional networks [6, 14, 15] do include some studies on KT-related phenomena (e.g., diffusion, knowledge transfer); however, the majority of their content centered on the study of social interactions that have implications for organizational functioning (e.g., friendships, work task assignments, staff recruitment, social support trust), but were not linked directly to the exchange or application of evidence to inform practice. Similarly, the emphasis on outcomes related to work satisfaction, leadership roles, professional behaviors, protocol efficiency, patient flow, operating room layout, technology adoption, and workplace performance reduce the extent to which KT-specific outcomes can be explored. The review by Chambers et al. [6] described primarily the settings and outcomes of these studies, whereas the current study aims to describe in detail the nature of the application of SNA to the study of KT. Such an approach aims to advance the science of KT by providing insight into worthwhile methodological directions this literature can provide. Evidence for the effectiveness of specific KT interventions or for the identified relationships between network properties and other variables relevant to KT can be sought elsewhere. Furthermore, none of these reviews examined the use of theory in their included body of literature specifically, despite this focus being an identified gap [6]. Given the rapid growth of the use of SNA in the health care context over the past 8 years (see Fig. 2), an updated and more directed search is warranted. A targeted examination of the research specific to SNA in KT and EIP is required to inform the application of SNA methodologies in this field, with attention paid to the insights offered by both the structural properties examined and the theoretical perspectives applied.

A SNA perspective can broaden our understanding of the mechanisms by which KT efforts are effective by examining the social structures and relationships that facilitate or hinder KT and EIP. This understanding will augment our knowledge base by expanding the range of KT determinants worthy of consideration. As researchers gain interest in the social drivers of KT and EIP, this review will provide a foundation for developing key research questions and SNA-driven methodological approaches for KT research that are based on established and relevant theories. Furthermore, this review will add to the current theorizing in the field related to a systems-focused understanding of KT and implementation processes. Specifically, implementation happens within a complex system, and network approaches have been used to study complex systems; the link between the two areas demands greater attention. Given these gaps, the purpose of this article is to synthesize the ways in which SNA methodology can be used to advance the science of KT.

## Methods

The purposes of a scoping review are to examine the extent, range, and nature of research in a given field; to determine the utility of conducting a subsequent systematic review; to summarize a body of research; and/or to identify research gaps, making it an ideal approach to map the KT literature utilizing SNA [16]. The specific objectives of this scoping review were (1) to describe the literature on SNA as it has been applied to KT and EIP involving health care professionals, in terms of its research design, methodology, and key findings; (2) to provide a critical analysis of the results in the context of existing theory; and (3) to identify strengths and gaps to inform future research. The scoping methodology, as described by Levac, et al.’s [17] modification of Arksey and O’Malley’s [16] guidelines, was applied. Step six in the methodology, consultation with key stakeholders, is optional and was not applied in the current review.

### Step 1: identify the research question

The specific research questions developed for this review were:

1. How has SNA been applied to health professional networks in the field of KT/EIP with respect to study aims, data collection and analysis methods, and populations, context, variables, and structural properties under study?
2. What are the primary theoretical underpinnings that explain the link between the network properties and KT/EIP?
3. What are the gaps in the literature that can inform future research directions?

### Step 2: identify relevant literature

The search strategy involved a systematic search of peer-reviewed English SNA literature in November 2015, repeated in July 2018, within five primary literature databases: MEDLINE, Cumulative Index to Nursing and Allied Health Literature (CINAHL), Embase, Web of Science (Science, Social Sciences Citation, and Arts and Humanities Citation Indexes), and Sociological Abstracts. Wherever possible, keywords were mapped to subject headings, which were focused to narrow the search (e.g., SNA terms) and exploded to broaden the search’s scope (e.g., health care professional-related terms) to best capture relevant articles. Keywords encompassed concepts related to social networks, KT, implementation and EIP, as well as health care professionals. Because of the lack of consistent indexing terms addressing the concept of KT [18], keywords and subject headings were drawn from empirically evaluated search strategies on this topic to foster relevant results [18–20]. The Appendix provides the detailed search strategy for MEDLINE; other database strategies are available on request.

### Step 3: select the literature

Retrieved articles were screened for inclusion by two authors (SG and EJ). Inclusion criteria included peer-reviewed articles describing outcomes of research studies employing quantitative SNA methodology to examine networks involving health care professionals in the context of KT, implementation, or EIP (broadly defined as the exchange and/or application of information to facilitate best practices in health care). The health professional context was selected to narrow the scope of the review while maintaining high relevance to KT, as health professionals are common knowledge users or subjects of implementation efforts. Outcomes of interest included, but were not limited to competencies (i.e., attitudes, knowledge, or skills) and behaviors by health professionals related to their sharing or use of evidence to inform clinical decision-making. Dyadic (i.e., pair-level), ego-network (i.e., individuals’ networks), and whole network (e.g., departmental or organizational-level) properties and variables were of interest. Exclusion criteria included non-English articles for feasibility, and articles that did not quantify SNA data or network properties to focus on articles that described, predicted, or explained network-related phenomena in the context of KT in quantitative terms specific to SNA (e.g., empirical studies whose analysis employed network data and analysis methods, as opposed to discussion papers). To target the scope toward evidence use by health professionals (i.e., to maintain relevance to KT involving health professionals within health care organizations), articles were excluded if they focused on online or social media-based networks (e.g., virtual communities of practice), policy-level KT, use of research by patients, focused on communication not explicitly involving research evidence or clinical decision-making about care based on evidence, or focused on the implementation of non-clinical interventions (e.g., electronic medical records, non-research-related quality improvement initiatives).

### Step 4: chart the data

SG extracted the data using a structured table developed a priori in accordance with the research questions. Information from each study was captured with respect to study aim, population, sample size, variables, KT process and structural properties examined, theoretical perspective and data collection, and analysis methods employed. A second reviewer (AK) screened the extracted data for accuracy.

### Step 5: collate, summarize, and report results

Descriptive statistics, including frequency counts and percentages, were calculated to provide an overview of the literature’s breadth. Articles were charted by year of publication and country of first author to illustrate the chronological and geographical development of the field. Each network property identified in the reviewed articles is presented with respect to the relational parameter it represents in the context of studying KT. Key findings related to network properties were compiled and summarized narratively. An analysis of the use of SNA and the theory that informed this body of research was then performed.

## Results

### Publication characteristics

**Fig. 1:**
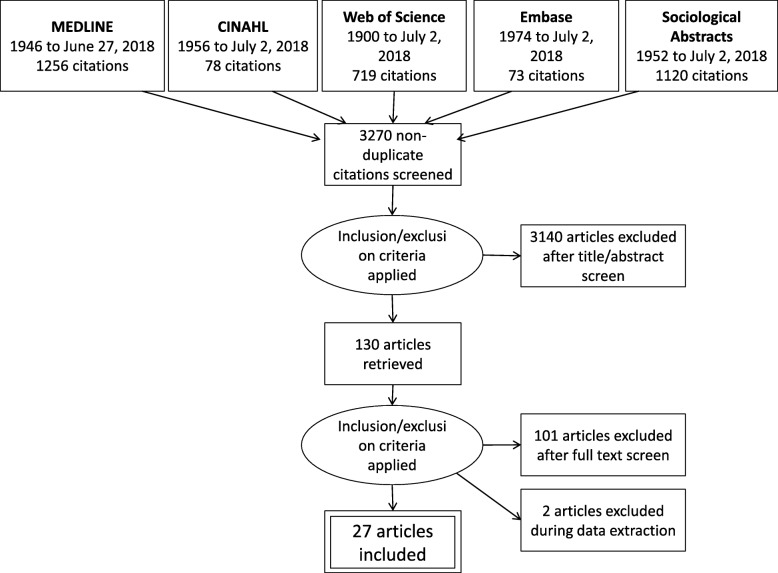
Flow diagram of the article screening process

**Fig. 2:**
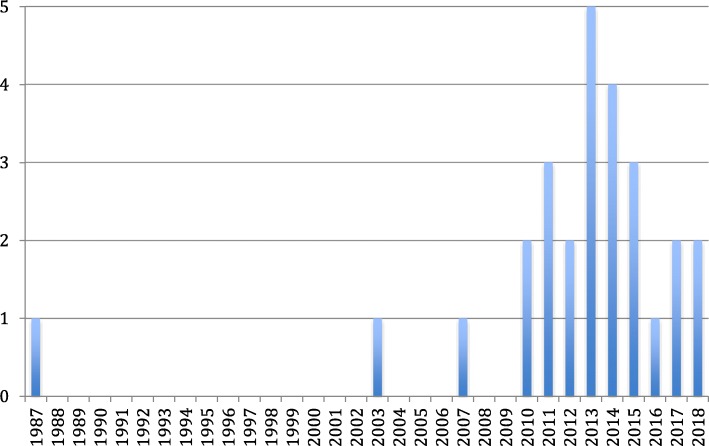
Publications by year

A total of 3531 articles were retrieved, of which 27 met inclusion criteria. Figure 1 shows the PRISMA flow diagram of included and excluded studies. Figure 2 depicts the frequency of publications by year. The USA and Italy (eight each) led in frequency, with Canada (4), Australia (2), the Netherlands (2), the UK (2), and Sweden (1) following. Study characteristics are presented in Table 2. Tallies and proportions of these studies presented in the following paragraphs do not sum to 100% in cases where the categories of characteristics are not mutually exclusive (e.g., a study may employ visualization of network properties while also presenting descriptive network property values).

**Table 2:** Characteristics of included studies

Citation	Study purpose	Type of network/setting	Network size (# participants)	Data collection methods	Theoretical perspective
Zappa 2011 [29]	To describe relationships for knowledge sharing about a new drug	Physician network within a group of 338 hospitals	784 physicians	Survey	Diffusion of innovation
Yousefi-Nooraie 2014 [37] (same study as 29)	To assess factors associated with information seeking in public health	Information seeking, expertise recognition, and friendship networks within an urban public health department	15 managers and 13 professional consultants (*n* = 28)	Survey	Transactive memory theory; social exchange theory
Yousefi-Nooraie 2012 [36] (same study as 30)	To identify the structure of intra-organizational knowledge flow for evidence informed practice	Information sharing network	170 directors, managers, supervisors, consultants, epidemiologists, practitioners, and administrative support		Social influence theory
Tasselli 2015 [22]	To describe knowledge transfer between professions, effectiveness of central actors and brokers, and the influence of organizational hierarchy on access to knowledge	Knowledge transfer network in a hospital department	*n* = 118 53 physicians and 65 nurses	Survey and interviews	Sociology of professions theory; SNA paradigm
Sibbald 2013 [21]	To explore patterns of information exchange among colleagues in inter-professional teams	Information seeking and sharing networks within six interdisciplinary primary health care teams	*n* = 28 (nurses, physicians, residents, allied health professionals, e.g., nurses, dietician, social worker); two sites: *n*_1_ = 19 and *n*_2_ = 8.	Survey and semi-structured interviews	SNA paradigm
Racko 2018 [47]	To examine the influence of social position on knowledge exchange over time	Knowledge exchange networks within three academic-clinical KT programs	three surveys: *n*_1_ = 66; *n*_2_ = 70; *n*_3_ = 42 clinicians and academics	Surveys	Social capital theory
Quinlan 2013 [42]	To explore mechanisms of information sharing across professional boundaries	Knowledge contribution to decision-making by members within multidisciplinary primary healthcare teams (two clinical decisions, so two networks for each of four clinical teams)	Nurse practitioners (*n* = 13 or fewer)	Online survey	Habermas’ theory of communicative power
Paul 2015 [35] (portion of data from study [45])	To test a model examining the role of triadic dependence on reciprocity and homophily	Influence network	33 physicians	Surveys	SNA paradigm
		Patient care network	135 physicians		
Menchik 2017 [44]	To explore the type of knowledge valued by physicians and the influence of hospital prestige on evidence-seeking behavior and perceived esteem by peers	Information seeking and clinical case discussion networks, within six hospitals	126 physicians	Survey	Social influence theory
Mascia 2018 [27]	To explore theoretical mechanisms explaining network formation across clinical sectors	Advice-seeking networks within two regional health authorities	97 pediatricians	Survey	Balance theory; structural holes perspective; homophily principle
Mascia 2014 [33]	To explore the association between connectedness with colleagues and frequency of evidence use within a physician network	Collaboration networks within 5 health authorities	104 pediatricians	Survey	Diffusion of innovation; social influence theory; social contagion; strength of weak ties [82]
Mascia 2015 [48] (same data as study [30, 32])	To explore the influence of homophily on tie formation				
Mascia 2011a [30] (same data as study [32, 48]	To determine the association between attitudes toward EIP and network structure and to identify predictors of collaborative ties	EIP advice-sharing networks within 6 hospitals	297 physicians	Survey	Homophily principle
Mascia 2013 [32] (same data as study [30, 48])	To explore the relationship between attitudes toward EIP and network position				Core/periphery model; structural holes
Mascia 2011b [31] (same study as [30, 32, 48])	To explore the association between network structure and propensity to adopt EIP		207 physicians		Social contagion; structural holes perspective
Long 2014 [41] (same data as study [40])	To examine the influence of clustering on past, present, and future collaborations within a translational research network	Past, present, and intended collaboration networks within a research network	68 researchers and clinicians	Online survey	SNA paradigm
Long 2013 [40] (same data as study [41])	To identify key players within a research network, their common attributes, and their perceived influence, power, and connectedness	Research collaboration and dissemination networks within a research network			
Keating 2007 [45]	To describe the network of influential discussions among physicians and to predict network position	Frequency of influential conversations relevant to practice within primary care	38 physicians	Survey	SNA paradigm
Heijmans 2017 [26]	To explore relationships between network properties and quality of care	Information exchange networks within 31 general practices	180 health professionals (physicians, residents, nurses, pharmacy assistants, social workers)	Survey document review (i.e., intervention and referral charting)	SNA paradigm
Guldbrandsson 2012 [38]	To identify potential national opinion leaders in child health promotion	Discussion network within national child health promotion context	153 researchers, public health officials, pediatricians and other individuals	Emailed survey item	Diffusion of innovation
Friedkin 2010 [25]	To examine the association between discussion networks, marketing, and physician prescribing practices	Advice and discussion networks of physicians (re-analysis of Coleman, Katz and Menzel, 1966 historical data on medication adoption)	125 physicians	Document review (i.e., prescription records of pharmacies)	Diffusion of innovation; social contagion; cohesion; structural equivalence
Burt 1987 [24]	To test social contagion theory by examining cohesion versus structural equivalence as drivers of tie formation				
Doumit 2014 [34]	To identify opinion leaders and their impact on EIP	Advice networks of craniofacial surgeons within 14 countries	59 craniofacial surgeons	Online survey	Diffusion of innovation
Di Vincenzo 2017 [28]	To explain the impact of research productivity on tie redundancy (i.e., connections that lead to the same people/information)	Advice seeking networks within and external to a health authority containing 6 hospitals	228 physicians	Survey	Structural holes perspective; homophily perspective
Bunger 2016 [43]	To evaluate change in advice ego-network composition and its impact on whole network structure following implementation of a “learning collaborative” model in improve care quality	Advice networks of clinicians (psychologists, social workers, others) and leadership in 32 behavioral health agencies	132 clinicians, supervisors and senior leaders	Surveys	SNA paradigm
Ankem 2003 [23]	To understand communication flow and its influence on awareness/adoption of a treatment, and to identify opinion leaders with influence	Frequent discussion networks within a sample drawn from an online physician directory	32 interventional radiologists	Phone interviews	Diffusion of innovation; SNA paradigm
D’Andreta 2013 [39]	To compare the network structures of three research/KT program initiatives	Informal advice giving and seeking networks within each of three academic-clinical KT programs	*n* = ~ 260 (directors, managers, program leaders, knowledge brokers, researchers, and others unspecified)	Online survey	SNA paradigm; Epistemic differences perspective

*SNA* social network analysis, *EIP* evidence informed practice, *KT* knowledge translation. Where indicated by the articles’ authors, the dependent variable is designated using bold text

### Study design and data collection

The 21 studies’ data sets were described in 27 articles, all but 2 of which employed cross-sectional designs yielding data at single time-points. Seventeen (81%) of the studies collected SNA data through surveys, 2 (10%) using a survey alongside interviews to support SNA data interpretation [21, 22], and 1 (5%) through telephone interviews [23]. Two (10%) studies employed document review (i.e., prescription, referral, or intervention records); one for SNA data collection and one to support outcome measurement [24–26].

### Networks and actors

Physician-only networks were the most commonly studied (11, 52%) [23–25, 27–35], followed by interprofessional networks of researchers and clinicians (6, 29%) [36–41]. Only one study (5%) examined the network of nurses and physicians [22], and one a network of nurse practitioners [42]. Two of the interprofessional networks included public health officials [36–38], one included leadership (i.e., directors, managers) and administrative support personnel [36, 37], and one included leadership and knowledge brokers [39]. In some cases, interprofessional network members’ professions or formal roles within the network were not clearly indicated [21, 38, 39]. For two studies, the analysis of a subset of network members (i.e., managers/professional consultants; physicians with specific clinical workloads) was carried out [35, 40].

Studies examined networks ranging in size from 13 to 784 participants, with a mean of 153. Just over half the studies (12, 57%) were conducted across organizational boundaries [23–28, 30–35, 43]. Eight (38%) were conducted within a single health care organization [21, 22, 29, 36, 37, 39, 42, 44, 45], one (5%) was conducted within a research-focused network [40, 41], and one (5%) within a health-specific field at a national level [38].

### Study purposes and data analysis methods

A SNA perspective has been used to explore the patterns and efficiencies of information sharing within and across professions, to identify positions of influence and determine their effectiveness, to predict or to explain patterns of ties based on attributes or network structure, to compare the structural characteristics of different models of KT, and to examine the relationship between network properties and EIP attitudes and behaviors. Using a longitudinal approach, SNA has been used to evaluate the influence of social structure on knowledge exchange over time, as well as to evaluate network change following a quality improvement intervention.

The majority of articles focused on information flow, while individuals’ adoption of a clinical practice, involvement in collaborations that support KT or EIP, and evidence-informed group decision-making garnered less interest. No research was available on other KT activities, such as the processes involved in developing guidelines or other KT tools, adapting knowledge to the local context, assessing barriers to change, facilitating departmental- or organizational-level practice or service delivery changes (including de-implementation), monitoring evidence use, evaluating KT effectiveness, or sustaining change over time [46].

#### Describing networks

Eight (29%) of the 27 articles described networks by deriving network properties from relational data [21, 22, 30, 36, 39, 40, 42]. Two articles (10%) used conventional descriptive statistics (e.g., frequency counts, proportions) to describe social network data [34, 38]. Network visualizations illustrated the data in 13 (48%) articles [22, 30, 32, 34–37, 40, 42–45]. Of these, ten articles presented whole network graphs (i.e., illustrations of the network structure), two presented network graphs of subgroups within the network, and one mapped whole networks within graphs that accounted for covariates. Network properties represented in the graphs included centrality (i.e., connectedness, brokers (i.e., bridgers)), core versus periphery structure (i.e., central areas of high connectivity versus peripheral areas of lower connectivity), and tie strength (e.g., frequency of contact); and attributes, such as gender, professional role, size of clinical practice, and division, department or team, and organization or site. Visualizations were used to depict network property configurations (i.e., illustrated the definitions of network properties) in two articles. Conventional charts (e.g., boxplots, scatterplots and bar, line, and area charts) were also used in six articles to visualize relationships between variables (e.g., network properties with one another, diffusion or adoption over time, centrality versus adoption timing, or receipt of useful information, percentage of ties by strength at different time points).

#### Testing hypotheses (network modeling)

Fourteen articles described the use of regression, including ordinary least squares [24, 28, 32, 44, 47], ordinal logistic regression [33], multi-level modeling [25, 26, 37], P2 logistic regression [35, 45], linear regression [22], and MR-QAP analysis [30, 48]. Regression was used to test theory about the cause of social structure [24, 25, 28, 35, 48], to explain the impact of social structure on knowledge exchange behaviors [47], ease of knowledge transfer [22], receipt of useful knowledge [22], or quality of care [26], and to describe the influence of hospital prestige on evidence-seeking behavior and perceived esteem by peers [44]. Three articles reported paired *t* tests or Wilcoxon ranks to evaluate differences between groups [22, 26] or time points [43], the latter of which also employed analysis of variance. Two articles described the use of the Chi-square test to examine associations between attributes and network position, or between two attributes [23, 40]. Exponential random graph models were used in three instances to predict or to explain the formation of ties based on attributes and network structure [27, 29, 37], and a single study employed factor analysis to construct groupings of individuals based on frequency of information exchange [23]. No studies employed stochastic actor-based network modeling to examine network change over time.

Sample hypotheses relating to tie formation included predictors, such as homophily, existing ties (leading to reciprocity), and having a formal mechanism within the organization for interacting. Further hypothesis examples included that higher professional status would be associated with more knowledge exchange, tie homophily (i.e., sharing the same profession with a connection) would be associated with greater knowledge transfer ease, the presence of brokers (bridgers) would be associated with an increase in the receipt of useful information, particularly to managers, and that greater connectivity, frequency of contact, homophily, the presence of a highly connected clinical coordinator, and being an opinion leader would be associated with an increase the use of best practices. For more information about the full array of correlational, dependent and independent variables and covariates (both relational variables and attributes) identified in the included studies, refer to Table 3.

**Table 3:** Variables, network properties and key findings

Citation	Primary data analysis method	Variables of interest			Findings
		Attributes	Structural or relational parameters	Network property used as proxy for structural parameter	
Descriptive/exploratory studies					
Yousefi-Nooraie 2012 [36] (same study as [37])	Deriving network properties to describe the network	_	Connectedness	Whole network density	Low density (1.2%) observed
			Information exchange	Tie reciprocity	Head management division identified as central cluster bridging organizational divisions, with hierarchical information flow. Expertise recognition and information seeking clustered within divisions; friendships spanned departments; Friendship and expertise recognition predicted information seeking ties; Network-identified brokers should receive same interventions and supports as formal brokers
			Prestige (key actors)	Indegree centrality	
			Mediating power of actors	Betweenness centrality	
			Subgroups of connected actors	Clusters	
			Brokers (actors connecting distinct teams/clusters of alters)	Brokerage patterns (measured by which groups the information source amd its recipients belonged)	
Sibbald 2013 [21]	Deriving network properties to describe the network	–	Cohesiveness related to giving and seeking research-related information	Whole network density	Low density for information seeking and giving (7–12%) observed; suggested these behaviors not a central focus of the interprofessional relationships
		Profession	Key players (prestige) in giving and seeking research information	Indegree centrality	Medical residents prominent in knowledge exchange; physician seen as primary implementer of evidence; nurses as intermediaries between physicians and support staff; allied health more likely to draw information from external networks
Quinlan 2013 [42]	Deriving network properties to describe the network	Profession tenure of the team Occupational distance among members Number of team members	Communicative power (i.e., the facilitation of mutual understanding among other team members)	Flow betweenness centrality	True interprofessional decision-making attributed to low structural hierarchy. Nurse practitioners (in newly formed teams) and registered nurses (in established teams) tended to have greatest communicative power. Mutual understanding and professions’ involvement varied across clinical decision-making episodes
				Change in flow betweenness centrality between clinical decisions	
Long 2014 [41] (same study as [40])	Descriptive SNA; correlations among specific network properties and/or attributes	Geographic proximity profession (e.g., clinicians/researchers)	Grouping based on similarity in attributes	# components	Geographical proximity, professional homophily associated with clustering (past collaborations); geographical proximity and past collaborative ties influenced current and future collaborations. Intended future collaborations were more interprofessional. Network density varied across networks (current 4%, future 27%, past 31%). Weak ties and reputation associated with intention for future collaboration; strong ties associated with current collaborations.
				External-internal (E-I) indices based on tie homophily	
				Clustering coefficients (comparing ego-and whole network density)	
		–	Past strong collaborations; Current or future collaborations	Past collaboration network tie strength; Current or future collaboration ties	
		Previous experience in the field	Current or future collaborations	Current or future collaboration ties	
		Actor’s reputation	Indirect contacts	Future collaboration network tie strength	
			Future intended collaborations	Future collaboration ties	
Long 2013 [40] (same study as [41])	Chi square analyses to test for association between attributes and network position (i.e., key actor status)	Current workplace Gender Membership in other networks Qualifications Approach to work within the network	Key actors (with respect to power, influence or connectedness)	Indegree centrality Betweenness centrality	A manager, and specific researchers and clinicians identified as key players. Apart from expert status and valuing adequate network resources, network-identified central players and formal brokers had little in common. Planned interventions and support for formal broker roles may be misdirected if not also offered to network-perceived central players or brokers. Network members may be better able to correctly identify central actors than formal brokers
Guldbrandsson 2012 [38]	Traditional descriptive statistics, e.g., frequency counts, percentages	Profession Work organization Geographical region	Information seeking about child health promotion	Tie homophily (%) Indegree centrality (frequency of being named)	Organization and professional field were shared in nearly half of all information seeking ties
Doumit 2014 [34]	Percentage of people nominating an actor; descriptive statistics (frequency counts, percentages)	–	Influence by central actors	Degree centralization	Six individuals with high credibility influenced 85% of the network, suggesting opinion leaders have potential for supporting evidence use
		Reasons for change in medical approach (proportions)	–	–	
		Barriers to clinical decision-making (proportions)	–	–	
D’Andreta 2013 [39]	Deriving network properties to describe the network (descriptive SNA)	KT model adopted	Prestige within the network	Degree centralization	KT teams with different models of KT (i.e., focus on research dissemination vs. knowledge co-production and brokering vs. integrated research-clinical collaboration) varied in their structural properties (e.g. the prominence and control of leaders in KT processes)
			Control over knowledge	Betweenness centralization	
			Access to knowledge	Closeness centralization	
			Alternate paths for knowledge flow that circumvent central actors	Flow betweenness centralization	
		Organizational role (e.g., director, support staff)	Core actors—dominant individuals with frequent knowledge exchange	Coreness scores (core-periphery algorithm)	
Predictive/explanatory studies					
Zappa 2011 [29]	Descriptive SNA	External communication (# visits from drug representatives); research orientation (# publications); clinical experience; hierarchical position (administrative role) Medical specialty* Hospital affiliation*	Colleagues with whom knowledge is discussed and transferred	Network density	Low network density (0.3%)
				Components	Multiple small components suggested lack of strong opinion leaders to drive treatment adoption. Findings suggest physicians tend to build small, closed, non-hierarchical internal, and external connections within their professional group, potentially limiting broader access to new information Isolates tended to be clinically experienced and active in research. Advice sharing more likely if physicians shared a medical speciality, geographic proximity but differed in research productivity or years in practice.
	Exponential Random Graph models (p* models)		*Tendency to exchange information with a number of sources*	Alternating k-stars	
			*Tendency to share knowledge within a small peer group* (*network closure*)	Alternating k-triangles; alternating independent two-paths	
			*Tendency to interact with similar others* Prominence as a knowledge source in the network	Tie homophily/hierarchy; indegree centrality	
Yousefi-Nooraie, 2014 [37] (same study as [36])	Descriptive SNA		Relative connectedness of actors of a given role	Indegree centrality Outdegree centrality	Managers identified as key brokers in KT interventions and EIP implementation processes. Public health professionals preferred to limit advice seeking and expert recognition to a small number of peers; advice seeking limited to own division. Strong friendship ties a significant predictor of information seeking ties. EIP scores not predictive of information seeking or expert recognition ties.
		*Role (e.g., manager) *Organizational division Score on EIP implementation scale	Key individuals	Degree centrality	
		Organizational division	Tendency to connect to peers from other units	E-I index	
		–	Tendency to reciprocate expert recognition and information seeking ties	Tie reciprocity	
	Exponential random graph modeling (ERGM)	*Role (e.g., manager) *Organizational division Score on EIP implementation scale	Tendency to connect with those with similar attributes	Tie homophily	
			Reciprocity	Tie reciprocity	
			*Formation of information seeking and expertise*-*recognition ties*	Ties and direction of ties (in vs. out)	
			Tendency for network to have highly connected nodes (hubs)	Alternating in-k-stars Alternating out-k-stars	
			Friendship connections	Ties	
	Multilevel logistic regression	*Role (e.g., manager) *Organizational division Score on EIP implementation scale	Tendency to connect with those with similar attributes	Tie homophily	
			*Formation of information seeking and expertise*-*recognition ties*	Ties	
			Friendship connections	Ties	
Tasselli, 2015 [22]	Paired *t* test Linear regression	*Gender *Tenure *Profession *Organizational unit *Rank (i.e., leadership role)	*Ease of knowledge transfer* *Perceived receipt of useful knowledge* Connectedness Hierarchy Network fragmentation Individual reach Brokerage potential *Network size	*Tie strength* Mean degree centrality Bonacich centrality Mean betweenness centrality Closeness centrality Betweenness centrality *Degree centrality	Knowledge tends to transfer within rather than across professions; nurses’ networks were denser and more hierarchical; closeness centrality positively associated with ease of knowledge transfer; brokering positions increased access to useful knowledge
Menchik 2010 [44]	OLS regression	# medical literature database searches per month # journals read regularly *Age *Gender *Tenure at hospital *Medical school *% clinical time *Sub-specialization Prestige of hospital (published rankings)	*Relational esteem by colleagues*	Indegree centrality	Physicians in higher prestige hospitals were less likely to be named as advice givers. Prestige in these settings associated with medical school attended. In lower-prestige hospitals, regularly reading a range of journals, and less time spent on clinical work increased likelihood of high esteem by colleagues
Mascia 2014 [33]	Ordinal logistic regression	*Self*-reported frequency of EIP use *Gender *# patients in caseload *area of clinical practice (e.g., asthma, urology, etc.) *# article subscriptions *perceptions of barriers to availability of evidence *perceptions of difficulty applying evidence to practice *Organizational affiliation *Affiliation to formal groups *Collaborative nature of actor’s medical practice	Degree of collaboration with colleagues	Outdegree centrality	Degree centrality directly associated with physicians’ EIP use
Mascia 2018 [27]	Exponential random graph models	*Past task force involvement *Tenure *Gender *Geographic distance *Association members *Health district	Tendency to reciprocate advice Tendency to seek advice from an indirect tie Tendency for local, generalized exchange of advice *Tendency to form advice ties *Popularity as an advice source *Advice-seeking activity *Brokering	Tie reciprocity Transitivity (path closure) Cyclic closure *Density *Indegree centrality *Outdegree centrality *Formation of non-closure structures *Tie homophily (of attributes)	Advice ties unlikely unless reciprocated; advice ties tended to organize around clusters—driven by transitivity, not popularity; Tendency against exchange of advice in cyclic structures; positive relationship between ties and association homophily in one health authority, and between ties and district/task forces in the other; tendency toward homophily related to tenure and distance, but not gender
Mascia 2015 [48] (same data as [31], [30] and [32])	Multiple regression-quadratic assignment procedure (MR-QAP)	Age Gender Tenure/seniority	*Frequency of collaboration* Similarity of professional role, institution and geographical location	*Tie strength* Tie homophily	Ties more likely if specialization, institution were the same between individuals; less likely if similar roles, greater difference in time since graduation and further geographic distance; professional homophily better predictor than institutional homophily
Mascia 2013 [32] (same data as [48], [30] and [31])	Descriptive SNA	Age* Gender* Hospital tenure* Tenure in health authority* Managerial role* Geographical distance from colleagues* Affiliation with other organizations* Self-reported EIP adoption (i.e. frequency of database searching)	Connectedness	Whole network density	Low density (5.7%) observed
	OLS regression		*Network authority* (*i*.*e*., *importance*—*relevant and popular)*	Hubs and authorities centrality	The most active EIP practitioners likely to be found at network periphery (i.e. least central)
			*Degree of coreness in the network*	Network coreness score (degree centrality and core-periphery position)	
Mascia 2011b [31] (same data as [48], [32] and [30])	Ordinal logistic regression	*Tendency to adopt EIP* (*self*-*reported frequency of peer*-*reviewed research use*) Age* Gender* Tenure in health authority/organization* Managerial role* # publications* Perceived access to evidence* Hospital affiliation*	Extent to which a given tie is redundant because of concurrent ties with another alter	Ego-network constraint	Physicians with greater network constraint (i.e., many redundant ties) reported decreased EIP adoption. May be related to information bias—tendency of physicians to interpret the information in a way that is congruent with their previous knowledge or opinion. High degree centrality associated with EIP use.
			Individual’s network size*	Total # of ties in ego-network (indegree + outdegree centrality)*	
Mascia 2011a [30] (same data as [32, 48])	Descriptive SNA	–	Average number of advice exchange colleagues	Mean ego-network density	Advice sharing most likely when physicians shared a medical specialty, geographic proximity, similar attitudes toward EIP, or had co-authored publications. Collaboration less likely when actors held similar managerial roles, or were at different hospitals/clinical/geographical areas.
			Tendency for colleagues to both give and receive advice with one another	Tie reciprocity	
	Multiple regression quadratic assignment procedures (MR-QAP analysis)	–	*Advice exchange among pairs of physicians*	Ego-network ties	
			Similarity between pairs of tied actors in: Geographical distances Gender* Age* Medical specialization* Clinical experience* Tenure in health authority/organization* Managerial role* # publications* Co-authorship* EIP adoption (self-reported frequency of peer-reviewed research use)*	Tie homophily	
Paul 2015 [35]	Extended p_2_ model with Bayesian modeling and estimation	*Age Patient age Patient sex Patient race Patient health status Patient intensity of care	Relative # shared patients *Same gender *Same specialty *Same location Reciprocity Social dependence (clustering)	Density Tie homophily Tie reciprocity Alternating independent two-paths Transitive triads Alternating k-stars (two-stars)	Low network density (0.10) observed. Triadic clustering higher than chance. Ties not associated with gender/specialty homophily. Location positively associated with ties. Complementary expertise positively associated with patient sharing. Transitivity may account for reciprocity
		Gender Clinic % female patients Self-identify as expert # clinics per week # years practicing in the city Tenure at hospital Years clinical experience Location of training	*Involvement in influential discussions*	Whole network density	Low density (0.154) observed; reciprocity more likely than not—may be an artifact of transitivity; high triadic clustering observed; same clinic and gender, expert, higher clinical caseload increased tendency for tie formation
Keating 2007 [45]	P2 logistic regression analysis				Self-identified experts seen as more influential; no relationship between # years in practice or location of work or training. Clustering with respect to EIP knowledge exchange observed between those with greater # of patients and higher frequency of clinical sessions. High reciprocity observed in the absence of opinion leaders with high centrality.
			*Being perceived as influential*	Indegree centrality	
			*Perceiving others as influential* (*information seeking*)	Outdegree centrality	
			*Reciprocity*	Tie reciprocity	
Heijmans 2017 [26]	Paired sample *t* tests/Wilcoxon tests Logistic multi-level analyses	*Patient age *Patient sex *Patient group *Patient illness status *Treatment/control group for parallel randomized controlled trial	Connectedness Frequency of contact Influence of coordinator Similarity in attitudes related to treatment goals Presence of opinion leader *Network size	Density Tie strength Degree centrality Homophily (E-I index) % of possible in-degree ties	Low density (0.37 and 0.38) observed. Most ties between those who did not value achieving treatment goals. General practitioner most likely named as opinion leader. Nurse performance associated with consistently identified opinion leader. Lack of tie homophily for positive attitudes associated with poor clinical outcomes
Friedkin 2010 [25]	Random intercept multi-level event history model	Professional age Chief or honorary position (yes/no) Number of journals read Value keeping up with scientific developments Physicians’ adoption of a new antibiotic (i.e., prescribing behavior) Marketing patterns of drug companies Proportion of previous adopters at a given time (“internal contagion”)	Influence of advisors/discussion partners	Contact network role (CNET)—a summative measure of 4 measures of structural cohesion and structural equivalence; position in the medical advice network	Cohesion and structural equivalence were correlated, and may be useful in combination to improve reliability in the evaluation of network structures across settings
Di Vincenzo 2017 [28]	Ordinary Least Squares regression	# publications *Tenure *Managerial role *Geographic distance *Hospital affiliation *# publications from same-specialty colleagues	*Dependence on others*/*access to new information* Relative productivity among ego-network colleagues Same role Same specialty	*Ego*-*network constraint* Euclidian distance *Ego-network size	Young employees appeared to have more redundant networks (greater need for advice). Hospital affiliation (i.e., context) influenced constraint. Constraint negatively associated with ego-network size and relative productivity (mediated by professional group membership), positively with productivity, Euclidean distance, role/specialty homophily (augments impact of productivity on prestige)
Burt 1987 [24]	Ordinary least squares regression with likelihood-ratio chi-squared test	Timing of adoption Relative timing of adoption within the network Professional age Contact with drug company Number of journals read Number of house calls vs. office visits Value keeping up with scientific developments	Position in the medical advice/discussion network	Structural equivalence	Adoption by others in equivalent positions within the network was a stronger predictor of adoption than adoption by those in an individual’s advice or discussion networks. Early adopters tended to participate in a range of EIP behaviors. Adoption by prominent physicians seen to be related to their desire to avoid being late adopters.
			Influence of advisors/discussion partners	Structural cohesion	
Ankem 2003 [23]	Chi-square statistics	Preferred information source Timing of awareness of the intervention *Timing of intervention adoption*	–	–	Clinical networks were most prominent in fostering awareness and adoption of a clinical intervention, but research and social networks also likely to influence these processes. Early adopters tended to rely on journals and conferences for information informing practice change; late adopters to a greater extent by network contacts.
	Factor analysis	Specialization Hospital City Timing of adoption Frequency of communication with colleagues	Types of relations within the network (e.g., clinical, research, leisure)	Ties	
			*Groupings of information exchange relations*	*Cliques*	
Longitudinal evaluative studies					
Racko 2018 [47]	Ordinary least squares regression	Professional status (ranking) *Professional role *Gender *Education *Organizational status	Research collaboration Joint decision-making Connectedness to high-status individuals Connectedness to knowledge brokers Connectedness to unfamiliar peers *Intra-professional whole network size	Ego-network size via tie heterophily Tie strength Mean status score of ego-network relative to whole network % of possible ties *Tie homophily	Higher social status associated with more research collaboration at all time points, and joint decision-making in early phases. Higher-status ties with peers, ties to formal knowledge brokers and ties to unfamiliar peers inconsistently predicted knowledge exchange, research collaboration and joint decision-making over time. Formal knowledge broker presence may facilitate interprofessional networking
Bunger 2016 [43]	Paired *t* tests; one-way analysis of variance; descriptive SNA	Role (faculty expert, internal colleague, external peer, private practitioner, other)	Connectedness Lack of advice seeking/sharing Reciprocity Similarity in connectedness Tendency for sub-groups to form Same institution Frequency of communication	Density Isolates Tie reciprocity Indegree centralization Clustering Tie homophily Tie strength	Ego-network size decreased, more markedly for senior leaders. Exposure to private practitioners and “others” decreased; exposure to experts increased. Substantial turnover in dyads was reported, with greater tie density around central core of experts. Reciprocity and tie heterophily increased over time

*KT* knowledge translation, *EIP* evidence informed practice. Where indicated by the article’s author, italic text = dependent variable; * = covariate

Information seeking patterns varied across professions and networks, with health professionals from some disciplines having a tendency to form small, closed subgroups, while others demonstrated greater connectivity and reach within the network, increased hierarchy (e.g., reliance on “gatekeepers” of information spreading it in a top-down approach), or relied more on sources of information or support external to the network (e.g., other organizations). Available information suggests that isolated individuals and those less connected at the periphery of the network may have more clinical experience and be more evidence-based in their practices than those at the network core [29, 32]. In the absence of a core-periphery structure (i.e., a more highly connected center with a less connected network periphery), degree centrality (i.e., the number of connections an individual has) may be a key factor associated with EIP use, at least for physicians [33]. A network-identified broker or opinion leader may increase access to useful knowledge [14], improve practice performance [26], and facilitate networking across professions [47].

### Network properties

Table 3 summarizes the key variables under study, the network properties that were derived from relational data, and the relational parameters (i.e., the constructs for which the network properties were acting as proxies). For example, network density was used as a proxy for connectedness in one study, and for representing the number of shared patients in another. Attributes of interest (which included individual characteristics, such as profession and gender; environmental characteristics, such as organization; social attributes, such as perceived reputation; and KT-related measures, such as EIP attitude scores) are also presented to offer a summary of the nature of non-SNA variables that have been analyzed alongside network properties. Study findings are presented in the final column for interest.

Eleven articles explored only a single or pair of network properties. Although 28 network properties were identified during data extraction, the majority of authors examined centrality, tie characteristics (e.g., the directions of the interactions; similarity in characteristics among pairs of connected individuals), and density (i.e., the proportion of ties relative to all possible ties) as their network properties of interest. Tie homophily (i.e., similarity of connected individuals on a given attribute, such as gender), indegree centrality (i.e., the number of people naming an individual as being connected to them), whole network density, the presence of ties, and tie reciprocity (i.e., bi-directionality in reported interactions or connections) were the most prevalent structural properties studied. The study authors’ discussions about the influences of these network properties were clearly linked to prominent theoretical perspectives. Less emphasis was placed on the analysis of centralization (i.e., the evenness of the distribution of connections), subgroups (i.e., groups of connected individuals not connected to other groups within the network), and transitivity (i.e., patterns related to sets of three individuals and their tendencies to share connections with one another).

Attributes, such as research versus clinical productivity, professional field or specialty, leadership role and organizational prestige [29, 30, 35, 38, 44, 48], as well as the presence of other types of ties (e.g., friendship, expertise recognition, previous collaborations) [30, 36], appear to be predictive or explanatory factors for the formation of information seeking or research collaboration ties. Conflicting findings regarding the influence of EIP attitudes, experience, gender, and geographical proximity on tie formation were identified [27, 30, 35, 37, 45].

### Use of theory

Diffusion of innovation was the theory most frequently applied (seven articles) [23–25, 29, 33, 34, 38]; social contagion theory [24, 25, 33] and social influence theory [33, 36, 44] were used in four and three studies, respectively. A model combining transactive memory theory and social exchange theory [37], as well as Habermas’ theory of communicative power [42], social capital theory [47], sociology of professions theory [22], balance theory [27], and an epistemic differences perspective [39], were applied in one instance each. Most commonly, theory was used to select network properties to examine and to develop hypotheses to test. In addition, theory was used to provide background information about SNA or the topic under study, to assist in the interpretation of findings, and to develop and test new analytical methods to advance the field of SNA.

Several other articles employed SNA-specific theoretical perspectives to exploratory analysis, including applying Granovetter’s strength of weak ties perspective [33], examining the association between structural holes (i.e., areas lacking connections) and the establishment of brokers that bridge network gaps [27, 28] or between a lack of ties and EIP attitudes [32], examining the role of social pressure on tie formation [24, 25], exploring the influence of being within a highly connected core of the network versus a less connected peripheral area on attitudes toward EIP [32], and evaluating network dynamics relative to the homophily principle (i.e., the tendency of people to form connections with similar others) [27, 28]. Seven articles employed a SNA paradigm without reference to a specific theory [21, 26, 35, 40, 41, 43, 45].

## Discussion

### Publication characteristics

While SNA has a long history in fields, such as sociology, mathematics, and psychology, its popularity in health care has shown momentum since the early 2000s [6]. Annual publication trends suggest an emerging interest in SNA as an approach to study KT processes and determinants since 2010. This concentration follows work by Thomas Valente and colleagues in the mid-late 2000s. Their work applied SNA in health care to identify and to evaluate brokers and opinion leaders as a means of promoting behavior change, linked communication networks and diffusion principles to health promotion research, and applied network principles to the study of community-based cancer research [49–51]. Increasing interest in the utility of SNA across a range of health care contexts, and the relative maturity of this field, may contribute to its continued use in the newer field of KT to embed new practices across settings.

### Study design and data collection

More longitudinal research would allow us to determine the direction of the causal relationships between network structures and attribute variables, such as EIP attitudes and behaviors, for better prediction of implementation outcomes. Such research would also enable the evaluation of changes in network connection patterns over time. This approach can be used to assess the impact of network interventions. Network interventions can use socially based strategies to identify and target network gaps [52] (e.g., interactive forums to help establish connections for isolated individuals or groups) to enhance KT processes, organizational capacity, or adherence to desired behavior (e.g., through social influence) [53]. Network interventions may also harness strengths in a network [52, 53] (e.g., engaging highly connected individuals to exert influence or to share resources) to better mobilize EIP attitudes, behaviors, or information flow for sustained implementation.

The simulation investigation for empirical network analysis (SIENA) framework offers a means of evaluating whole network dynamics, particularly with networks defined by connections that persist over time, which could be particularly helpful in identifying barriers to the successful introduction of interventions in specific settings [54]. The SIENA framework can evaluate change in repeated measures of a network with respect to actors and ties, as well as the interplay of these network changes alongside changes in the behaviour of network members [54]. For example, a network intervention aimed at reducing isolation within the network, or at increasing the connectedness of individuals who are positioned well to influence many others, can be evaluated for its effectiveness using the SIENA framework. The evidence of these changes would serve as supporting mechanisms for new evidence-based interventions. Computational models, such as agent-based modeling, can also be used to represent individuals and their interactions. Multiple simulation experiments that are programmed based on attribute data and structural characteristics allow researchers to specify and to control the parameters of the computational algorithms in order to determine the effects of specific variables [55].

Survey use was the dominant method of SNA data collection employed, which is consistent with the broader SNA field [7]. Although interviews were not prominent in the reviewed articles, they can gather the same relational data as surveys, while allowing the participant and interviewer to clarify question and response meaning (e.g., defining operational terms in more depth; describing the reasoning behind naming specific people in one’s network) [56]. A qualitative data collection approach may have implications for the size of the sample, but the opportunity for discussion can increase response validity, while the relational network data can be quantified for analysis [56]. Qualitative interpretive strategies can also be used to understand context and meaning within the network and the phenomenon of interest [56], such as the extent to which network structure and/or specific attributes or contextual factors are perceived to influence attitudes, knowledge, or behavior related to the introduction of a new intervention. Mixed methods can be used to triangulate findings to validate results, and to strengthen the explanatory power of the research by exploring the complexities involved to a greater depth [56].

Limited use of document review was also observed, none of which involved electronic data. The use of secondary data (e.g., email records, social media interactions, medical records) to quantify networks may be more or less time intensive than primary data collection; limitations may also exist in the types of variables available to study [7]. However, particularly with electronic data, documentation of network activity may be readily available through regular quality monitoring, and span a time period to enable longitudinal analysis [7]. For example, data related to evidence sharing communication patterns and subsequent use of best practices by health professionals (e.g., intervention approaches, treatment intensity, or dosage) within and across clinical teams can be leveraged to identify network strengths and gaps, and to monitor KT strategy effectiveness.

Observation is a fourth means of SNA data collection, which was absent from the reviewed studies. Although more resource-dependent and not without risk of observer-influenced behavior change, observation may enable the identification of ties not captured through self-report [7]. For example, interpersonal dynamics during a meeting may be recorded by a third party more objectively than meeting participants may recall, while concurrently focusing on the content of the meeting. Self-report data also presents potential bias related to recall, particularly when respondents are asked to think back to interactions in the past, or to report their frequencies [7]. While network rosters can be used to help mitigate this problem in clearly bounded networks (e.g., an organization), in larger networks this strategy can create excessive burden on respondents [7]. Careful attention to the way questions are worded, and consideration of the number of alters requested of respondents must be made to gather meaningful and accurate data [7].

### Networks and actors

With more than half of the included studies examining physician-only networks, and only a handful studying interprofessional health care teams, great opportunity exists to expand the range of professions under study. Because of the growing shift in health services delivery from profession-based to collaborative practice models involving interprofessional teams [57], further research is needed to evaluate the generalizability of findings beyond physician networks, as well as in other health care contexts. The diversity of network sizes and settings, however, demonstrates the utility of SNA for a broad range of applications, from the study of interventions in small hospital health care teams to large multi-organizational or national networks. The examination of inter-organizational networks (i.e., organizations as nodes) in the context of KT was beyond the scope of this review. Further study may be warranted to scope out this literature and to determine its implications for informing KT from an organizational network perspective.

### Study purposes and data analysis methods

The examination of information exchange processes, key players, reasons for tie patterns, associations between network properties and various attributes, and the evaluation of KT intervention outcomes are crucial for understanding how to successfully embed a new practice. However, because of its examination of information flow, predominantly, this body of research presents a narrow view of KT that focuses primarily at the individual level of evidence-based decision-making. This limitation relates in part to the scope of the review, as well as the consideration that other actors (e.g., health leaders, researchers) rather than health professionals may typically manage many of the KT activities that were not represented. Extending the application of SNA to broader organizational or group processes and through a wider range of KT-related activities and actors will advance our understanding of the network dynamics involved in all aspects required to move evidence into action. Such phenomena of interest may include the collaborative production of KT tools, barriers assessments, implementation processes, and evaluation efforts. Examining these processes from a network perspective has the potential to identify strengths and gaps in the network that need to be addressed, to explore the structural characteristics and associated attribute-level variables that might contribute to their success, to describe the role of health professionals in these processes, and to evaluate network-level KT interventions to facilitate them.

The limited number of network properties (i.e., three or fewer) examined in more than half of this body of literature suggests that the potential for greater SNA-related insights from these studies to inform future research and practice in KT remains largely untapped. Simply describing networks or examining a single network property (e.g., tie homophily, centrality) and its association with attribute variables fails to leverage SNA’s full potential. As KT scientists, we are interested in not only what is happening, but why it occurs, and the processes involved. With this information, we are positioned more effectively to design network-based KT interventions.

For example, basic SNA can be used to identify key players with influence within the network; subsequent analyses can be used to explain how these individuals came to hold these positions. Knowledge of an individual’s structural position may also help to determine from whom they may seek evidence or KT support. This information can be used to develop KT interventions that target specific health professionals or groups of individuals based on their network structure or key attributes to strengthen KT processes. For instance, influential individuals can be leveraged as champions or knowledge brokers to improve the efficiency of information exchange or behavioral influence within a discipline group. Individuals with attributes in common with key players can be selected to lead KT interventions within an interprofessional health care team. Alternate paths for efficient information exchange or behavioral influence can be accessed if resistance by specific individuals is encountered.

An understanding of relational influences can also advance the science of KT by improving the specificity of KT interventions, and by supporting their evaluation. For instance, KT intervention fidelity (e.g., intended versus actual information flow) can be monitored using SNA, and the KT intervention can be adjusted accordingly over time to address gaps or barriers. Network-specific outcomes of a KT intervention (e.g., increased connectedness, access to information) can also be evaluated empirically based on relational data. Collaboration between KT and SNA researchers may enable a more in-depth examination of the data available from KT research, to bring new insights from a network perspective.

Visualizations are an asset in SNA research because of their ability to represent the data in a way that makes it more accessible to those less familiar with SNA methodologies [58]. Surprisingly, fewer than half of the articles presented network maps, which suggests that researchers could do more to elucidate descriptive relational findings for readers. While more complex than descriptive analyses, graphing the results of p_2_ models can illustrate the relationship between binary network data and covariates, while factoring in network structure [59]. Stacked correspondence analysis of matrices representing different time periods can be used to visualize network data at different time points [7]. Supplemental graphing using conventional visualization methods (e.g., bar, scatterplot, line charts) is also available as an approach to visualize the relationships among network properties and attributes that has yet to be fully leveraged. Appropriate visualization methods and techniques must be selected to answer the research questions of interest, while preserving clarity [7, 60].

While a range of analytical techniques was identified, many studies employed traditional analytical techniques designed for data meeting assumptions of independence. By their nature within the SNA paradigm, dyadic data do not meet these assumptions. Techniques designed to account for interdependencies in the data, including quadratic assignment procedure (QAP) analysis and exponential random graph models (ERGM), are considered more robust for those analyses of specific hypotheses involving dyadic ties or network characteristics. These approaches enable the modeling of relationships between dyadic (i.e., relational) variables (e.g., information exchange) and attribute variables (e.g., gender), between dyadic variables (e.g., similarity in EIP attitudes, and engagement in research collaboration), or at the whole network level (e.g., density of communication ties relative to time to evidence adoption) [7]. An example from the included literature is the use of ERGM to help determine whether particular individuals—say those with similar personal characteristics—are connecting for information sharing more than expected due to chance [29].

With the inclusion of longitudinal designs, analysis approaches, such as stochastic actor-based network models (SABM) that examine network change over time, can begin to be represented in this body of literature. SABM can represent both ties and individual attributes to examine network change. As an example, Yousefi-Nooraie et al. [61] used SABM to determine the effect of their intervention (evidence-based decision-making skills) on participants’ status as knowledge brokers.

### Network properties

Further KT-related research that includes analyses of centralization, subgroups, and transitivity may afford a more in-depth understanding of the network-related influences on KT among health professionals. Centralization (i.e., the unevenness of connectivity across the network) can be calculated for the whole network, or for departments or sectors within an organization for the purposes of comparison. Subgroups (e.g., smaller connected groups within a network) can be identified and addressed individually during a KT intervention. For example, isolated individuals can be engaged to form connections with colleagues to benefit from their knowledge or influence. Different subgroups may receive different KT interventions based on their characteristics and what evidence or theory suggests their influence might be. Efforts to link or to expand subgroups may precede implementation efforts to establish an environment more conducive to change.

Transitivity has been used to examine the tendency of individuals to exchange information with a small versus a large number of sources, and for the network to form highly connected hubs. This analysis can inform the design of KT interventions to improve the efficiency of information sharing or influence (e.g., identifying targets for the intervention and relying on transitive processes to spread the information rather than targeting all network members). Such a strategy can then be compared to alternatives, to test hypotheses about the influence of different network properties on the effectiveness of KT interventions. This evaluative work is critical to improve our understanding of network influences on KT processes and outcomes.

The range of structural properties examined suggests that researchers consider multiple structural phenomena to be relevant to KT processes and outcomes. Considerable overlap also existed in the real-world phenomena being evaluated by proxy through these properties. This diversity provides a foundation on which to build a stronger knowledge base about networks’ multiple influences on research use. This approach aligns closely with current discussion in the KT literature about complex health systems, and the need to use “complexity-informed approaches” to embed evidence-informed changes in the health care system [62–64]. Such systems models assume that health care organizations are dynamic, interdependent, contain sub-systems with feedback loops, and exhibit emergent properties. A combination of a complex adaptive system lens and SNA modeling to measure and explain features of networks and individuals, and most critically the relationship between networks, sub-networks (like cliques), and individuals as they change over time, is an underutilized approach to KT and implementation. The approach moves from a mechanical understanding of KT barriers and facilitators to a much more complex picture of what is required to introduce and to sustain change in health care organizations.

While complex statistical models are more difficult to apply, they present the benefit of multivariate analyses to better examine the interaction between various factors, as well as the opportunity to control for covariates that may be inflating or masking key effects. As statistical models for SNA continue to emerge, these tools will become increasingly important in clarifying the relative influence of various network properties and attribute effects thought to influence KT.

An understanding of the influences of individual attributes and different types of ties (e.g., friendship) can support the structuring of the health care environment to strengthen network density within homophilous groups (e.g., discipline groups), or to foster greater diversity of collaborations within the network (e.g., interprofessional clinical, project, or professional development work). Knowledge of network structure can be used to target EIP behaviors using a social influence approach that introduces innovation into the network’s core or brokers to reduce pervasive information bias, or that facilitates greater density overall. Unsurprisingly, different approaches to KT (e.g., leader-driven vs. collaborative; researcher-led dissemination vs. researcher-clinician collaboration) may present different structural properties (e.g., hierarchical vs. clustering). This finding has implications for information flow, as well as for the development of network interventions to address gaps or to leverage prominent actors in the network to champion the innovation. Those with formal health care leadership roles may not be the only individuals with influence; informal brokers may be more recognizable by network members (particularly peers) as central to KT processes than formal leaders [37, 40]. Alternatively, some networks may not present with prominent central actors or opinion leaders for EIP [29]. A SNA lens can assist in identifying these individuals when they exist, analyzing the extent of their reach, determining the reasons for their prominence, and developing a network-informed plan to leverage their position to advance KT.

Normative group processes and structural position can also explain the timing of adoption, which may be useful in identifying early adopters, opinion leaders, and shared attitudes within a network [23, 24]. Differences reported in the preferred information sources and network influences for early versus late adopters from a diffusion of innovation perspective can be used to guide differential approaches to behaviour change.

### Theoretical insights

Various theories, frameworks, and models guide KT research efforts [5], although only in a small proportion (3–6%) of primary research articles in the broader KT field [65, 66]. The included articles demonstrated a broad range of theoretical approaches drawn from the fields of sociology and psychology (e.g., diffusion of innovation, social influence, social contagion, social exchange), as well as from the field of SNA itself (e.g., perspectives that explain the role of weak ties, structural holes, cohesion, or tie homophily on network dynamics). The variety of approaches used suggests that diverse theories may merit exploration for their utility in KT-related SNA research, and that multiple theories may be applied in a single study (e.g., [24, 25, 33]).

Diffusion of innovation theory is applied commonly in the KT literature [5], so its frequent application here was not unexpected. The theory’s principles lend themselves well to a SNA paradigm, in that the theory was developed to predict or to explain how information or innovation spreads within social systems [67]. The collection of attribute data about network members permits the analysis of characteristics that influence an individual’s adoption of innovation, relative to their network position and other contextual factors [68]. While the majority of KT strategists have adopted an educational approach (i.e., by implementing KT interventions based on an “information dissemination paradigm”) [5, 69] to improve awareness, understanding, and attitudes as a means of influencing uptake [70, 71], the role of social networks in their impact has yet to be comprehensively studied. Studies that pair traditional educational and behavioral outcomes research with SNA may provide greater insight into the social mechanisms that influence these outcomes.

Social contagion/influence theories are common in the SNA literature, and purport that actors share attitudes, knowledge, or behaviors because of their ties to others who influence them [72]. This perspective highlights the role of peers and others in fostering behavior change, attitudes, and identities [69]. The study of opinion leaders, audit and feedback [5, 66, 71], and mentoring [73, 74] may follow a social influence perspective [69], as does a growing body of research on knowledge brokering as a human mediator of research uptake in health care [75]. Social capital theory, another prominent SNA theory, attributes tie formation to an individual’s need for social capital from others (e.g., resources, information, power). This contrasting approach was used in the context of knowledge brokering; its application in tandem with social influence theory may provide insight into the direction of causality (i.e., ties form because of attributes vs. attributes are the result of ties) [7].

Transactive memory theory, social exchange theory, Habermas’ theory of communicative power, sociology of professions theory, balance theory, and an epistemic differences perspective have logical applications to SNA. Each of these perspectives addresses social factors applicable to networks. The transactive memory-social exchange model describes how information seeking behavior is influenced by the awareness and valuing of another individual’s knowledge or skills, their accessibility, and the cost or effort involved in seeking the information [37]. Communicative power is meant to describe the influence of a third party on the mutual understanding achieved between a pair of individuals [42]. Sociology of professions theory describes how members of a shared profession tend to develop a collective status based on their unique health care jurisdictions, common training, and mutual knowledge, which can limit their tendency to interact outside this peer group [22]. Balance theory contends that individuals tend to develop balanced relationships (e.g., tie reciprocity) in order to circumvent unease [27]. An epistemic differences perspective purports that individual attributes in combination with structures, processes, and other features of the environmental context (including power), influence individual performance and experiences [39]. These differences create diversity within the network that generate opportunities for novel sharing and innovation [39]. By understanding the structural network factors that contribute to information seeking, epistemic differences, power dynamics, and communication processes, we may gain a more holistic understanding of KT. Further analysis is required to determine the utility other theories and frameworks from the KT and SNA literature to SNA-related KT research.

The study of networks is particularly relevant when considering the role of social evaluation in the diffusion of innovation. Gartrell [76] argues that social evaluation is a key driver of decision-making, noting the role of networks in providing norms and comparison opportunities that influence behavior. Social influence theory, and the principles of tie homophily, social contagion, and structural equivalence parallel this line of thought. While the role of context is addressed relatively commonly in KT and implementation models and frameworks [75], the concept of social evaluation is largely absent from the non-SNA KT literature. SNA can be applied to identify the attributes, structural positions, or nature of the relationships that are most influential for KT processes from a social evaluation perspective, including the role of brokers, homophily, hierarchy, centrality, and other network properties. These network characteristics offer insights into the patterns and restrictions in the flow of information as well as into power structures, key actors, and their reach and efficiency. Normative group processes and structural position can also explain the timing of adoption, which may be useful in identifying early adopters, opinion leaders, and the development of shared attitudes within a network [24, 76].

Social constructivist and cognitive learning theories have also emerged in the KT literature to explore the means by which health professionals interact within their social context to construct and to understand knowledge [66, 77, 78]. The network perspective has not been combined with these approaches to enrich our understanding of the role of this social context on evidence uptake. Also missing from the included articles is reference to behavior change theories that have been prevalent in the KT literature [5]. These theories emphasize the various barriers and facilitators to change, including personal, organizational, and system-level factors [65]. Integrating a SNA lens to behavior change models may augment their utility by including social structure as a determinant of behaviour change.

### Limitations

The search and screening process used in this review limited inclusion to studies involving quantitative analysis of SNA data; a greater depth of understanding about SNA’s utility for KT may also be gained from theoretical discussion papers. Also omitted was the contribution of qualitative data from network research, which can provide insight into how social structures, network properties, and the nature of relationships influence KT from the perspectives of network members. A broader set of inclusion criteria that encompassed studies on the adoption of non-clinical best practices, including technologies, training strategies, quality improvement initiatives and other innovations, as well as KT in the policy context, may also inform aspects of KT research and implementation initiatives, but was beyond the scope of this review.

This discussion only addressed these studies’ SNA-specific findings. Important non-SNA-related findings (e.g., the most important sources of evidence to augment awareness and adoption [23]) have not been summarized but can also inform KT research and practice. In addition, the scope of this paper prevented an in-depth discussion of the full breadth of theoretical perspectives represented in the SNA KT literature, which may warrant a separate review. Finally, because of the variability in study designs, the lack of inclusion of quality appraisals in scoping review methodology, and the inconsistency of network properties examined across the studies, results should be interpreted with caution until further research can evaluate their quality and generalizability.

## Conclusions

Given the diversity and complexity of health professional KT networks, optimal strategies for KT may vary depending on the structure of a professional or organizational network, as well as on professional identities and personal attributes. Within a given setting, interprofessional dynamics, hierarchy, social influence, centralization, brokers, and other important structural properties are worthy of consideration. The SNA paradigm offers a broader lens by which to examine and to influence KT processes and outcomes across contexts, while drawing on established theories known to the KT science field. SNA extends the scope of KT influence to include social relationships and structural characteristics of individual and whole networks. However, its full potential has yet to be realized.

Suitable for relatively small (e.g., a dozen) to larger networks of several hundred members or more, SNA can be used to describe or to evaluate groups within or across departments, organizations, countries, or beyond. Longitudinal research, a more representative range of populations, the use of interviews, document review and observation for data collection, greater depth of analysis, and the leveraging of network visualizations can augment the contributions of SNA to the KT science knowledge base.

Understanding how network properties can be used as proxies to measure social processes (e.g., information exchange, best practice adoption, decision-making, influence) can help KT scientists to apply SNA effectively to expand the range of measures that can be used to evaluate KT efforts. The approach can be used to describe a network as a precursor to a KT intervention, as a means of supporting planning (e.g., identifying target groups or individuals), as well as for testing hypotheses. Evaluating information sharing, positions of influence, relationships between network connection patterns and individual attributes (e.g. attitudes) or behaviors, and the effectiveness of KT interventions relying on or targeting networks are all feasible. Predicting or explaining patterns of connections, comparing groups, time points or contexts are also possible.

Finally, while this article did not present a comprehensive overview of the use of theory across the entire body of SNA-related KT literature, it does offer a starting point for conceptualizing theory-based SNA applications in KT research. In keeping with a systems or complexity theory approach, SNA can offer a wider spectrum of determinants to examine in evaluating KT processes by addressing social factors.

The targeted SNA research outlined here may help to highlight the role of interactions, relationships, and other social dynamics throughout the full scope of activities and processes required to move evidence into action within health care settings and beyond.

## Appendix

**Table 4:** OvidSP MEDLINE search strategy

Concept	Keywords	MeSH Headings
Health care professionals	Clinician* OR “health care professional*” OR “health professional*” OR therapist* OR physician* OR doctor* OR nurse* OR “allied health”	Allied Health Personnel OR Medical Staff Hospital
Knowledge translation	Knowledge translation OR evidence based practice OR evidence informed practice OR dissemination OR organi*ational innovation OR implementation adj3 research OR research utili*ation OR research use OR knowledge utili*ation OR knowledge transfer OR knowledge mobili*ation OR knowledge exchange	Knowledge Management OR Translational Medical Research OR Diffusion of Innovation OR Evidence-Based Practice OR Professional Practice OR Guideline Adherence OR Social Change
Social network analysis	Social Network, social networks, network theory	Interprofessional Relations

## Abbreviations

CINAHL: Cumulative Index to Nursing and Allied Health Literature
EIP: Evidence informed practice
KT: Knowledge translation
SIENA: Simulation investigation for empirical network analysis
SNA: Social network analysis
